# ‘One-size doesn’t fit all’: Understanding healthcare practitioners’ perceptions, attitudes and behaviours towards sexual and reproductive health and rights in low resource settings: An exploratory qualitative study

**DOI:** 10.1371/journal.pone.0234658

**Published:** 2020-06-25

**Authors:** Gilbert Tumwine, Jack Palmieri, Markus Larsson, Christina Gummesson, Pius Okong, Per-Olof Östergren, Anette Agardh

**Affiliations:** 1 Social Medicine and Global Health, Department of Clinical Sciences, Lund University, Malmö, Sweden; 2 St. Francis Hospital Nsambya, Kampala, Uganda; 3 Centre for Teaching and Learning, Faculty of Medicine, Lund University, Lund, Sweden; 4 Health Service Commission, Kampala, Uganda; Università degli Studi di Perugia, ITALY

## Abstract

Although progress has been made to improve access to sexual and reproductive health services globally in the past two decades, in many low-income countries, improvements have been slow. Discrimination against vulnerable groups and failure to address health inequities openly and comprehensively play a role in this stagnation. Healthcare practitioners are important actors who, often alone, decide who accesses services and how. This study explores how health care practitioners perceive sexual and reproductive health and rights (SRHR) and how background factors influence them during service delivery. Participants were a purposefully selected sample of health practitioners from five low income countries attending a training in at Lund University, Sweden. Semi-structured interviews and qualitative content analysis were used. Three themes emerged. The first theme, “one-size doesn’t fit all’ in SRHR” reflects health practitioners’ perception of SRHR. Although they perceived rights as fundamental to sexual and reproductive health, exercising of these rights was perceived to be context-specific. The second theme, “aligning a pathway to service delivery”, illustrates a reflective balancing act between their personal values and societal norms in service delivery, while the third theme, “health practitioners acting as gatekeepers”, describes how this balancing act oscillates between enabling and blocking behaviours. The findings suggest that, even though health care practitioners perceive SRHR as fundamental rights, their preparedness to ensure that these rights were upheld in service delivery is influenced by personal values and society norms. This could lead to actions that enable or block service delivery

## Introduction

Although progress has been made regarding access to sexual and reproductive health (SRH) services in the past two decades, in many low-income countries, improvements have been slow. Discrimination against vulnerable groups and reluctance to address health inequities comprehensively play a role in this stagnation [1, 2]. Health care practitioners (HCPs) are central in enhancing accessibility. They possess information, skills and power, and are uniquely positioned to determine how health policies and guidelines are implemented to enable people to realise their health rights [3, 4]. While formal training empowers HCPs with knowledge and skills, and policy guidelines highlight the services to be provided, implementation of health interventions ultimately depend on the discretion of HCPs that interface with the clients [5]. As a result decisions on a particular health intervention may differ widely between HCPs within the same health system [6]. This is partially because HCPs’ decisions during service delivery may be based on their values and attitudes [7–10]. This gap between professional knowledge and decisions made during service delivery is a critical dimension that undermines equitable accessibility to services, especially in the sensitive areas of SRHR [11, 12].

In instances where rights are not respected, discrimination may occur or clients may shun services due to fear of stigma. The socially marginalised, such as sexual minorities, are especially susceptible to discrimination [13]. In Pakistan, for example, 22% of women reported fear of rude health workers as the second most common reason for not using contraception services [14]. In South Africa, 17% of women with post-abortion complications reported that staff attitudes had discouraged them from seeking a legal abortion at a government clinic [15]. In India and Tanzania, men who have sex with men, opted for self-medication or delayed seeking care because of stigmatising behaviours from providers [16, 17].

Although a number of strategies to address challenge have been examined, including beneficiary participation and advocacy for best practices [12, 18, 19], full appreciation of contextual influences on SRHR health care practitioners’ attitudes and behaviours in low-income countries is still evolving.

This exploratory study, drawing on insights from a purposefully selected sample from 5 low income countries, seeks to understand health care practitioners’ attitudes and behaviours towards sexual and reproductive health and rights (SRHR). Specifically, it explores how health care practitioners perceive SRHR, and how background factors influence them during service delivery.

## Material and methods

Participants in this study were enrolled in the 2016 international training programme (ITP) in Sexual and Reproductive Health Rights (SRHR) at Lund University, Sweden. ITP is commissioned by the Swedish International Development Cooperation Agency, aiming at improved health equity for marginalised populations [20]. The training programme focuses on SRHR as an understanding of human rights applied to sexual health, sexual rights, reproductive health and reproductive rights. ITP targets health care practitioners holding strategic positions in medical facilities and civil society organizations, both in private and public sectors with potential to facilitate improvement in service delivery in low and middle-income countries. The ultimate aim of ITP is to bring about organizational change. Implicit in this model is the belief that participation in ITP contributes to positive changes in participants’ knowledge, values and attitudes concerning SRHR that will precipitate a sustainable and equitable change in their professional behaviour.

In 2016, ITP participants were from Ethiopia, Zambia, South Sudan, Zimbabwe, and Bangladesh. Each country team consisted of 4–6 adult males and females. They included 5 midwives, 4 nurses, 6 doctors, 8 midlevel managers and 5 policy makers. Data collection took place in February 2016 before the participants started their intensive four-week training in Sweden. All participants were eligible to participate.

A qualitative research design with in-depth interviews was used. This study conformed to the requirements established by the Helsinki Declaration [21].

Each participant was approached individually by a researcher and provided with information concerning the aims of the study, its methodology, and assurance of privacy and confidentiality in the presentation of results. The information sheet also had the contact details of the responsible researcher. It was emphasised that participation in the study was entirely voluntary and that participation or non-participation in the study would not have any effect on their ITP training and that they were free to withdraw from the study at any moment if they so wished. The participants were then given time to read the information and ask questions before signing an informed consent form.

Two male interviewers, who were Lund University employees but not involved in the ITP, conducted the interviews. Both interviewers had prior experience and training in qualitative research methodology and master’s degrees in public health (MPH). One of the interviewers (GT) is an obstetrician and gynaecologist (MD). Both had experience working with SRHR in healthcare systems in low resource settings. Half the interviews were conducted by one interviewer, and the other half by the second interviewer. Briefing and debriefing between the two interviewers and the last author (AA)was done to ensure consistent data collection. The interviews were conducted in English in private rooms at Lund University and without any other person present. An interview guide with open-ended questions was developed and pre-tested on students attending an international masters program in public health at the university. All interviews were recorded and transcribed verbatim for analysis. The mean length of the interviews was 45 minutes (range 30–120 minutes). No names were collected, and interview participants were referred to as “P” and assigned numbers. No reimbursement was offered to the participants.

A manifest and latent content analysis was performed, according to the guidelines described by Graneheim and Lundman [22]. The transcripts of the interviews were chosen as the unit of analysis. Interview transcripts were read through multiple times to gain an overview of the content. From these transcripts, the first author identified words and sentences that were relevant to the study aim and extracted these as meaning units. These meaning units were condensed, while keeping the core of the text, and assigned codes. Codes were then grouped together into sub-categories on the basis of similarity of manifest meaning, and sub-categories were further aggregated into broader categories. Themes were developed based on the underlying meaning expressed in the categories. At each step AA was consulted to ensure consensus and coherence. Open code software was used to facilitate the coding procedure [23]. The final model, including codes, sub-categories, categories, and themes, was agreed upon by all co-authors. An example of the analytical process can be found in Table 1.

**Table 1:** An example of the analytical process moving from meaning unit to category.

Meaning unit	Condensed meaning unit	Code	Sub-category	Category
Well, I think of SRHR as the right that people have to enjoy with regards to their sexuality, sexual education, accessing of SRH services, and with regard to…what is in their countries’ constitution.	SRHR as a right to enjoy one’s sexuality, sexual education, access SRH services, and in regard with country’s constitution.	SRHR being rights to sexuality	Ability to make free choices being key to well-being	Rights being fundamental for SRH
		SRHR being rights to sexual education		
		SRHR being rights to access SRH services		
		SRHR varying with country laws	Local context determining application of rights	SRHR not existing in a vacuum

## Results

All ITP participants were approached and requested to participate in the study. To optimise variation in gender and country of origin, 28 were interviewed, resulting in 18 males and 10 females, with a mean age of 38 (29–57).

Three main themes emerged from the analysis. The first theme, **One-size doesn’t fit all in SRHR**, reflects health care practitioners’ (HCPs') perceptions of rights with regard to SRH. Although HCPs’ viewed rights as being fundamental for SRH, the exercise of these rights was seen as context-specific. The second theme, **Aligning a pathway to service delivery**, describes experiences influencing HCPs’ perceptions of and attitudes towards SRHR and how these are balanced on a regular basis when arriving at decisions. The third theme, **Health care practitioners acting as gatekeepers for SRHR**, illustrates the pivotal role HCPs play, either by blocking or facilitating service provision. These themes are supported by 5 categories and 12 sub-categories as shown in Table 2.

**Table 2:** Overview of the analytical model describing HCP’s perceptions, attitudes and behaviours towards SRHR.

Sub-category	Category	Theme
Ability to make free choices being key to well-being	Rights being fundamental for SRH	‘One size doesn’t fit all’ in SRHR
Fulfilment of SRHR being a shared responsibility		
Level of development determining level of rights	SRHR not existing in a vacuum	
Local context determining application of rights		
Perception of rights changing over time		
Possessing negative views against abortion and LGBT	Balancing between personal values, society norms and the rights-based approach	Aligning a pathway to service delivery
Aligning with society norms towards SRHR		
Holding onto professional ethics		
Accepting gender stereotypes that subordinate women	Navigating through gender stereotypes	
SRHR being seen as a women’s issue		
Lacking adequate knowledge to address LGBT health needs	Dealing with own knowledge gaps	
Being cognisant of own experiences with SRHR		
Objecting to restrictive social norms	Enabling access to SRHR services	HCPs acting as gatekeepers for SRHR
Leading by example		
Blaming the victim	Blocking access to SRHR services	
Upholding barriers in service delivery		

In the following section, the findings are presented by theme (in bold and underlined), with their respective categories in bold and sub-categories underlined. Quotes (shown in italics) have been added to support the understanding of the analysis and to ground the results in the words of the participants [24].

### ‘One-size doesn’t fit all’ in SRHR

This theme describes HCP’s perceptions of the different components of SRHR. Participants understood SRHR, as human rights applied to SRH. Under the category of **Rights being fundamental for SRHR**, having sexual and reproductive health rights was considered as crucial for one’s well-being and synonymous with human rights. Participants understood such rights as the ability to safeguard one’s well-being through unhindered access to service provision, a guarantee to optimal sexual health, and the Ability to make free choices being key to well-being:

*I think of SRHR as human rights…freedom of choice…rights that I need to have…no one should refuse me…people are supposed to enjoy…regardless of their sexuality or who they are*. (P9)

Sexual and reproductive health rights were perceived as entitlements equivalent to ‘…*being in a free space to do what you want*.*’* (P2), and making own decisions concerning one’s sexual and reproductive well-being. Enjoyment of rights was perceived to be universal irrespective of gender, ethnicity, race, or sexual identity.

HCPs considered realisation of rights as the responsibility of many actors, underscoring the essence of Fulfilment of SRHR being a shared responsibility. This responsibility was seen as shared among individuals, health care providers, and their governments and meant, for example, that individuals bear a responsibility to prevent unwanted pregnancy and sexually transmitted infections:

*I would want to believe that whenever a person engages in sexual activity*, *they know what will follow*. *I think it’s up to an individual to take care of the consequences…don't blame the doctors or government*. (P3)

Health care practitioners were expected to bear the responsibility of providing service equitably, free from discrimination while maintaining confidentiality and privacy, while governments were thought to be duty bound to safeguard these rights by guaranteeing a conducive policy and legal environment.

The category **SRHR not existing in a vacuum** describes the HCPs’ understanding of the ability to exercise SRH rights as something that should be considered within a particular context. Consequently, Level of development determining level of rights emerged as a sub-category. In this sub-category, participants regarded it as unrealistic to expect poor people to focus on LGBT rights or legalisation of abortion in communities lacking basic needs:

*We come from a very poor community that lack…clean water*, *roads*, *schools*…*so what is a priority for such a community… to go and preach about LGBT…or to raise a level of living standards and education*? (P5)

Promotion of rights was seen as competing for resources that would otherwise be used for development purposes, suggesting *‘developed countries are better at handling LGBT than poor countries*.*’* (P10)

Investment in development first, through education, was seen as a key catalyst for fulfilment of individual freedoms in SRHR. With regard to marital rape, for example, it was expressed that:

*In some societies*, *people are ignorant*. *Some of them accept that its part of life…but those who are educated now can actually talk to husband…most of the cases*, *in the villages*, *they just accept*. (P6)

It was generally thought that with development and education, acceptance of rights would automatically come.

Participants also believed that communities differed in their understanding and practice of SRH rights, with Local context determining application of rights, even within the same country. HCPs constantly perceived rights through a morality lens. This meant that some aspects of SRH rights were deemed unacceptable, depending on the moral values of the individual HCP or their community. Concerns arose when deciding whether acts that conflicted with core religious or culture beliefs should be considered as rights:

*There are some rights that are morally incorrect*…*for a woman to be carrying a condom*…*is morally upside down*. (P11)

More vivid views were expressed when discussing abortion and LGBT. Whether or not an individual had a right to terminate pregnancy, or whether an individual had the freedom to engage in non-heterosexual practices depended on how acceptable these issues were in their specific community. Most participants felt that, although individuals had rights, abortion and LGBT practices were immoral and unacceptable. A right to abort, for example, was equated with *‘having a right to terminate unborn life’* (P14), and the right to engage in LGBT sexual practices was regarded as an unnatural personal preference that was unacceptable to the community:

*In our society homosexuality is not something we accept as natural*. (P9)

Understanding the context also meant recognising that acceptability of some aspects of SRHR was dependent on a number of national and international actors, who influence decision-making and policy formulation and whose level of influence differed from place to place:

*This issue of same sex is more of a political issue than a SRHR issue*. . . . *European Union refusing to give country money because of LGBT*…*when it’s treated like that it becomes a political issue not a health issue…foreigner telling you to accept it*. (P6)

These context-bound differences were also seen as temporal, with communities said to learn and unlearn attitudes towards different aspects of SRHR. Thus, there was a Perception of rights changing over time:

*Sometime back we would say that its (homosexuality) a personality disorder*…*this time we are told it’s a preference*…*we respect what someone believes in*. (P6)

Despite the participants agreeing that rights are fundamental to SRH, they were also in agreement regarding the context-bound nature of SRH rights and the need to take this into consideration.

### Aligning a pathway to service delivery

The second theme describes the experiences influencing HCPs’ perceptions of and attitudes towards SRHR. It illustrates their personal journey, towards arriving at a position during service provision as a pathway that involved **Balancing between personal values, society norms and the rights-based approach,** being aware of **Gender stereotypes normalising inequality** and **Dealing with own knowledge gaps.**

The on-going internal struggle experienced by many HCPs arising from having personal values that might not align with the rights-based approach to the services that they were expected to provide is captured in the category, **Balancing between personal values, society norms and the rights-based approach**

The HCPs in this study were aware of Possessing negative views against abortion and LGBT. Some participants thought of abortion as killing the baby and a ‘western idea’. Although the participants also held strong negative views against homosexuality, they believed that there was no need to discriminate against them in service delivery, as reflected in this quote:

*My actual belief is that being an LGBT is a sin…but then who is not a sinner*…*what is right and what is wrong*. *(P12)*

The HCPs took refuge in their societies’ prevailing restrictive political climate or legal system to justify their own beliefs or attitudes, saying that they were simply Aligning with society norms towards SRHR. Existence of political or legal opposition to an aspect of SRHR seemed to provide a necessary external support to reinforce individual HCP’s beliefs:

*Same sex marriages are not legally permissible in our country…I am a law-abiding citizen*…*I don’t support homosexuality*. *I don’t see a reason why someone should be having sex with the same sex person*. *(P15)*

In contrast, other HCPs felt that the absence of an enabling law or political environment was a significant obstacle while working with sexual and reproductive health, as illustrated by the following:

*When I talked to parliamentarians about LGBT*, *they started labelling me homosexual*… *there is that fear of being judged as service provider*. (P4)

When conflicted between personal values and client expectations, HCPs were holding onto professional ethics or codes of ethics related to their training. The doctors’ oath, ‘to preserve life’, was frequently mentioned when deciding against abortion to preserve the life of the unborn foetus or providing abortion to save a woman whose life was in danger.

However, occasionally participants felt that their beliefs were inconsistent with what was professionally expected of them, accepting some aspects of SRHR while at work, which they disagreed with in private:

*We live a double life as professionals*. . . . *executing our work*, *we don’t have problems with SRHR*…*but this is not changing our beliefs*…*the way I speak to individuals outside work sometimes doesn’t reflect my professional life*. (P5)

While discussing individual and societal beliefs, the idea of **Gender stereotypes normalising inequality** re-occurred throughout the interviews. HCPs typically mentioned Navigating through gender stereotypes as a challenge to achieving SRHR equity. Men, and specifically husbands, were thought to have full responsibility over most SRH decisions, including when to have sex in a marriage, whether or not to use contraception, determining the number of children, or deciding when girls should get married:

*For my daughters their father has a right to say*, *you have to get married… even before she is supposed to get married*…*in most of our communities it happens like that*. *I can’t say no as a woman*. (P13)

Women were considered to be subservient and, in most cases, had limited or no say in many SRH decisions. As a result, HCPs were constantly confronted with situations where services were available, e.g. contraception, but women’s inability to make decisions hindered utilisation. This is reflected in the quote below, regarding whether legalising abortion would make it easy for HCPs to provide it or not:

*Our understanding of rights has little to do with this…her status in the community determines if a woman will come to hospital or not*. (P11)

Although gender stereotypes and inequality were thought to disproportionately affect women, it was also believed that perceiving SRHR as women’s issues distanced men from full participation. In many communities, men were not expected to be present in SRH facilities such as maternity wards and family planning clinics:

*When men go to clinics*, *they find lots of women and its very uncomfortable*. *It’s the culture I think that doesn’t consider men*. (R6)

Many health care practitioners had little or no knowledge in some aspects of sexual and reproductive health, and the category **Dealing with own knowledge gaps** describes the actions they took to address these gaps.

The main area in which the HCPs felt that they had a severe knowledge gap was when it came to issues of sexual orientation and same-sex sexual behaviours. Thus, Lacking adequate knowledge to address LGBT health needs was manifested through doubts over how it was possible to love a person of the same sex, what the purpose of such a relationship would be, how such couples derived pleasure, and how they could deal with children raised in same sex marriages. In response to this, most participants opted for self-education:

…*I had to buy a book*…*to understand why and how they end up being gay*…*I read how some decided to make it as a choice and others were born like that*. *It’s interacting with these people*, *it is getting knowledge and getting educated that aided this journey of understanding*. (P15)

Other health care providers found answers through social interaction:

*We spend time with friends who have strong views on these issues and we tend to think as a group…for me this plays a bigger role in me accepting the LGBT group of people and challenging my culture*. (P4)

Having lived through particularly unpleasant experiences seemed to inform and influence HCPs’ perceptions and attitudes towards a rights-based approach. Being cognisant of own experiences with SRHR thus appeared as an important factor when discussing knowledge gaps. These experiences included having faced discrimination because of gender or sexual identity, having had one’s privacy or confidentiality compromised while seeking care, or having dealt with unplanned pregnancy in one’s life, as illustrated by the following:

*At first I never understood why people decide to abort… but at one point… I stopped being rigid*. *For three years I was being treated for infertility*. . . . *but I never conceived*…*so I went to school*, *I found I was pregnant when I didn’t have any help*. . . . *I considered having an abortion because delivery was coinciding with exams*… *I wanted to withdraw from school*…*I had all these thoughts about terminating pregnancy*. (P7)

Making decisions during service delivery, therefore, was influenced by one’s attitudes and values, professional ethics and societal norms, in a web of gender stereotypes and own experiences. Almost on a regular basis, HCPs considered a combination these influences, which differed according to which aspect of SRHR was being considered

### Healthcare practitioners acting as gatekeepers

The third theme captures how HCPs’ perceptions of and attitudes towards SRHR affect their decisions (behaviour) towards SRHR services. Grounded in their own notion of individuals’ SRH rights, HCPs exhibited behaviours or actions that were either enabling or blocking access to SRHR

The category **Enabling access to SRHR** describes proactive actions taken by the HCPs to push for service availability in the face of resistance. One way in which this was achieved was through Objecting to restrictive social norms. This included advocating for youth access to contraception in communities where access was prohibited, campaigning against forced marriages where teenage girls were married off early in exchange for family gifts, or using education to promote tolerance for sexual minorities where they are discriminated against, as exemplified by the following:

*I ensure that LGBT are given an opportunity to access the health facilities without prejudice or discrimination*…*they are humans like me*. (P4)

Other HCPs enhanced access to SRHR services through Leading by example. These actions included prioritising SRH for young women, providing services without value judgments to sexual minorities, or helping young girls seeking abortion services who had been rejected by colleagues, as illustrated by the following statement:

*While working in a maternity ward that used to have a lot of young girls coming in with pregnancy*…*others would reject them*…*I would take time to make them comfortable*, *find out their concerns*, *counsel them…these are the people who need these services*. (P8)

On the other hand, the freedom to independently interpret the right to SRH services could also lead to HCPs **Blocking access to SRHR services** in their role as gatekeepers. Some providers adopted a Blaming the victim attitude, for instance regarding seeking repeated abortions as an act of *‘carelessness’* and an *‘abuse of service’* (P6) or deciding by themselves who would or would not receive services. Thus, some HCPs did not feel that LGBT persons would be welcome in their facility:

*It’s definitely a strong NO…from a service providers perspective*…*I wouldn’t welcome things (Homosexuality) like that at our facility*. (P15)

In other instances, HCPs would require that LGBT persons remained discrete about their sexuality if they were to receive care at a health facility:

*I am ok with them as long as they don’t throw it in my face*…*they don’t put it in my face*. *I’m ok with them being far away from me and not making it part of my problem or part of my life*. (P19)

Other practitioners denied or compromised access to care by Upholding barriers in service delivery. Some of the behaviours exhibited included practitioners deciding against a client’s need to terminate pregnancy and instead insisting that a pregnancy be carried to term and a baby be given up for adoption instead:

*Whatever the case*…*I will encourage to her to continue pregnancy*, *to give her options not to give up her pregnancy*. *I can never allow her to abort*. *Never*! (P2)

This was rooted in the belief that the life of the unborn foetus was sacred and had to be preserved. Healthcare practitioners were therefore able to leverage influence over access to services based on their own interpretation of the laws. This resulted in gatekeeper behaviour that enhanced access to services, but also subverted it.

## Discussion

To the best of our knowledge, this is the first study to explore access to SRHR services from the practitioner’s perspective involving HCPs from different low-income countries, with diverse cultures and religious backgrounds.

The main objective was to understand the decision-making processes among healthcare providers from Ethiopia, Zambia, South Sudan, Zimbabwe, and Bangladesh working with SRHR. Specifically, we sought to understand how HCPs perceive SRHR, what background factors influence their perception of, and attitudes towards, SRHR and what effect this perception had, if any, on their decision-making during service delivery in SRHR

Three themes emerged: **One size doesn't fit all in SRHR, Aligning a pathway to service delivery,** and **Healthcare practitioners acting as gatekeepers**. Together, these themes highlight the complex interplay between personal, cultural, and societal factors that shaped HCPs’ perceptions of and decision-making regarding SRHR. Although rights to sexual and reproductive health were universally understood as fundamental for all human beings, fulfilment of these rights was perceived to be dependent on the local context, varying with the level of development and changing over time. Healthcare providers were mindful of their own values, societal norms, gender stereotypes, work rules, and own knowledge gaps regarding SRHR, and constantly balanced these against one another on their route to service delivery. The resulting action, or inaction, of these healthcare providers, played a critical role in clients’ ability to access SRHR services.

### “One size doesn't fit all” in SRHR

Health care practitioners recognised SRH rights as fundamental for one’s wellbeing and synonymous with human rights. This was expressed as a desire for services free from discrimination, protection of freedoms of sexual identity and its expression, the guarantee of privacy, confidentiality for all persons—an understanding that is consistent with the rights-based international and national agendas [25–27].

In addition, participants expressed an understanding that the fulfilment of individual rights is a shared responsibility, an observation that fits with the rights-based approach, obliging duty bearers, such as the state and individuals in positions of responsibility, to promote and protect human rights and empower rights holders to know and claim their rights [27]. More recent consensus regarding the urgency to accelerate SRHR for all suggest that, it “will depend on individuals demanding for their rights, civil society organisations advocating on behalf of the individuals and governments respecting, protecting and fulfilling those rights” [2].

However, HCPs frequently perceived rights through a moral lens, accepting those aspects and dimensions of SRHR that were considered morally consistent with their own beliefs and societal norms, rejecting those that they considered morally inconsistent. As a result, even though there was a shared understanding of the rights concept in SRH, its application varied between the participants and differed depending on other societal factors. While reflecting on the global trends affecting SRHR, Starrs et al, [2018] acknowledge that achieving rights largely depends on broader social, economic, cultural and health care contexts which differ considerably.

The absence of legal and political protection of SRH rights in low-income countries was acknowledged as negatively influencing the implementation of these rights. This agrees with other studies that point to the legal recognition of human rights as one important precursor to protecting and promoting SRH rights in low-income countries [28]. In addition, it was generally believed that socio-economic development requires more urgent attention than SRHR in low-income countries. The observation that low socio-economic status negates accessibility to SRHR has been reported in other low-resource contexts [7–9].

The moralising attitude of healthcare practitioners has been reported in other studies [29–31], suggesting that health practitioners’ attitudes mirror the values of the larger societies to which they belong. This potentially leads to discrimination and reduced access to services for those discriminated against. What seems like a straightforward definition of SRH rights as universal, therefore, becomes more complex in the context of societal norms and personal beliefs, as reflected in the notion that ‘one-size doesn’t fit all’ in SRH.

#### Aligning a pathway to service delivery

Moving from understanding SRH rights to service provision turned out to be a complex pathway navigated between personal values, workplace and societal norms and attitudes, and an appreciation of knowledge gaps.

Participants in the study frequently reported deep-rooted negative attitudes against the lesbian, gay, bisexual and transgender community, as well as abortion—both seen as unacceptable in most circumstances. These attitudes appear to have been influenced by cultural and religious norms. These findings are echoed in other research concerning healthcare workers’ stigmatisation of men who have sex with men (MSM) in low-resource settings, where findings showed that religious and societal factors played a large role in this [17, 32].

The fact that the above-mentioned attitudes were commonly expressed opinions among the healthcare providers suggests that such societal norms might have become institutionalised in health care settings, thereby making it more difficult for vulnerable groups to access health services due to stigma and discrimination. Denying services to SRH based on practitioners’ beliefs has been reported in different forms, including the refusal to offer services, or demanding some form of authorisation before a service is provided [33, 34]. This may push individuals to seek services from unskilled personnel, leading to severe morbidities or even death.

Even when participants considered themselves neutral to either abortion care or LGBT health needs, they most often felt constrained to align with societal norms. Simpson et al. have reported this form of socialisation among nurses in training, suggesting that healthcare providers commonly tend to comply with attitudes and actions that co-workers find acceptable [8].

In addition to personal norms, professional ethics, codes of conduct, national laws and policies were a common reference point when deciding whether or not providing SRH services was permissible. The existence of policies and laws prohibiting certain activities was used to justify decisions taken to deny service provision to particular groups. In some cases, the reason given was moral, but the justification was legal. This has been observed in Uganda where the presence of legislation against homosexuality has been used to justify denial of service provision [35]. On the other hand, codes of professionalism and ethics for healthcare providers were used to justify provision of services that contradicted moral opinions of the service providers. This has been shown in other research, such as in Ghana, where adherence to professional ethics superseded moral objections towards providing abortion services [36].Thus, policies and laws were weighed against personal values and professional codes of ethic and decisions were taken based on this balancing act.

The participants in this study were aware that inequality, in accessing SRH services, mostly affected young women and sexual minorities but felt ill equipped to address this challenge. Inadequate preparedness of clinicians to address needs of sexual minorities has been reported in other studies [30, 31, 37–39]. Equitable access to SRHR services therefore should include training of healthcare practitioners on dealing with sexual minorities and other vulnerable groups.

#### Healthcare providers acting as gatekeepers for SRHR

Participants in this study held positions in their respective health systems that allowed them to make decisions in provision of SRHR services. They reportedly acted in ways that could be understood as gatekeeping, either to enable or block access to SRH services.

Some of the enabling behaviours included advocating for non-discriminatory access to services such as contraception and abortion services, and sexual health counselling. As mentioned above, in these situations the providers worked with the code of conduct or fought against the national laws to provide the services needed.

Understanding these pathways to positive provider behaviour, and how some providers are able to work against social norms whilst others are unable to do so, is of utmost importance for identifying change agents in SRHR. This is especially true for aspects such as abortion and contraceptive services where acceptance has been a challenge in many low-income countries.

However, other participants reported actions and negative attitudes that could potentially stand in the way of service access. Personal values, religious beliefs, and lack of supporting laws were common reasons why providers stated that they distanced themselves from services such as abortion care or dealing with LGBT health needs. This finding that is supported by previous research in low resource settings, such as studies concerning the continued influence of judgmental views of service providers in Uganda [8], and the role of cultural belief systems on client-provider interactions in low-income countries [9].

A systematic review of the effect of gatekeeping in healthcare delivery from the perspective of institutionalised processes reported that gatekeeping significantly lowers utilisation of health services [31]. Evidence of the form of gatekeeping reported in this study, that is provider dependent and non-institutionalised, is very limited.

### Methodological considerations

This study involved healthcare practitioners from Ethiopia, Zambia, South Sudan, Zimbabwe and Bangladesh, each country team consisting of both men and women with diverse religious and cultural backgrounds. The participants were midwives, nurses and doctors, middle level managers, and policy makers with different experiences in the field of SRHR. Although saturation was achieved earlier during the interviews, all the 28 consenting participants were interviewed to capture the diversity from the different countries, religions, cultures and gender. The inclusion of all participants with diverse backgrounds ensured variation and enhanced the credibility of the study finding [24].

To ensure consistency of the data collected, a semi-structured interview guide was used, frequent briefing, and debriefing took place between the interviewers, and extensive observation notes were taken during the interviews. This ensured that any new insights or sensitive issues arising from preceding interviews were shared and discussed before further interviews were conducted. The use of a table showing an example of the analysis process and the presence of dense description facilitate an audit trail.

Although the researchers in this study were themselves healthcare practitioners with potential personal biases and interests, extensive notes detailing the impressions and decisions made along the research process were made and frequently referred to, to ensure neutrality. Extensive quotations showing how the conclusions are ‘grounded in the data’ were employed.

The ITP participants were a purposefully selected group of healthcare practitioners from diverse cultural and religious backgrounds, at different levels of service delivery in low-income countries. Although they might be more “aware” than the average HCP in these settings in other countries, it is plausible that the valuable insights gained from this study may be transferable to healthcare providers working with SRHR in similar contexts.

The main limitation of the study is that the participants in this study were interviewed just prior to participating in the ITP at Lund University in Sweden, i.e. away from their normal setting. It is unknown if this had any influence on how they responded to the interview questions.

## Conclusion

The findings suggest that there is a disconnect between what the participants knew about SRH rights and their preparedness to ensure that these rights were upheld in practice. Although the SRH rights were acceptable as universal, their application depended on which SRHR component and required adaptation to individual or societal norms and economic settings. Training for healthcare practitioners needs therefore to provide opportunities for value clarification to help HCPs become more aware of how deeply rooted these attitudes are and how these can enable or block health services.
